# Multivalent peptidic linker enables identification of preferred sites of conjugation for a potent thialanstatin antibody drug conjugate

**DOI:** 10.1371/journal.pone.0178452

**Published:** 2017-05-30

**Authors:** Sujiet Puthenveetil, Haiyin He, Frank Loganzo, Sylvia Musto, Jesse Teske, Michael Green, Xingzhi Tan, Christine Hosselet, Judy Lucas, L. Nathan Tumey, Puja Sapra, Chakrapani Subramanyam, Christopher J. O’Donnell, Edmund I. Graziani

**Affiliations:** 1 Worldwide Medicinal Chemistry, Pfizer Global R&D, Groton, Connecticut, United States of America; 2 Pfizer Oncology Research, Pearl River, NY, United States of America; Helsingin Yliopisto, FINLAND

## Abstract

Antibody drug conjugates (ADCs) are no longer an unknown entity in the field of cancer therapy with the success of marketed ADCs like ADCETRIS and KADCYLA and numerous others advancing through clinical trials. The pursuit of novel cytotoxic payloads beyond the mictotubule inhibitors and DNA damaging agents has led us to the recent discovery of an mRNA splicing inhibitor, thailanstatin, as a potent ADC payload. In our previous work, we observed that the potency of this payload was uniquely tied to the method of conjugation, with lysine conjugates showing much superior potency as compared to cysteine conjugates. However, the ADC field is rapidly shifting towards site-specific ADCs due to their advantages in manufacturability, characterization and safety. In this work we report the identification of a highly efficacious site-specific thailanstatin ADC. The site of conjugation played a critical role on both the *in vitro* and *in vivo* potency of these ADCs. During the course of this study, we developed a novel methodology of loading a single site with multiple payloads using an *in situ* generated multi-drug carrying peptidic linker that allowed us to rapidly screen for optimal conjugation sites. Using this methodology, we were able to identify a double-cysteine mutant ADC delivering four-loaded thailanstatin that was very efficacious in a gastric cancer xenograft model at 3mg/kg and was also shown to be efficacious against T-DM1 resistant and MDR1 overexpressing tumor cell lines.

## Introduction

Antibody-drug conjugates have proven to be a promising new area of cancer therapy with over 50 ADCs in clinical trials and numerous more in the pre-clinical pipeline [1, 2]. Though the concept of targeted therapies like ADC has been around since the early 1900s [3], recent improvements in technology and our understanding of the science has led to three approved ADC drugs: MYLOTARG^®^, ADCETRIS^®^ and KADCYLA^®^. Despite the enthusiasm generated by these drugs, there is still much to be improved regarding their homogeneity, efficacy and safety [4, 5]. Both MYLOTARG^®^ and KADCYLA^®^ are generated by conjugating the cytotoxic payload to surface lysine residues, of which there are over 80 on a typical mAb, resulting in a complex heterogeneous mixture [6, 7]. Though ADCETRIS^®^ is generated by conjugating MMAE to the eight native hinge cysteines on an IgG1 antibody, the optimal ADC is still a stochastic mixture of species [8].

In order to improve the homogeneity of the ADC product, the field has been steadily moving towards site-specific conjugation approaches. This could result in improved manufacturability and ease of characterization [9, 10]. In addition, there have been multiple reports showing that site-specific conjugates may offer improved efficacy, stability, and safety in certain cases [11–14]. Some of these ADCs have started to percolate into the clinic, paving the way for the next generation of ADCs [15].

We herein report the development of a site-specific ADC conjugated to a novel class of cytotoxic payload, spliceostatin. Thailanstatin A, a natural product analog of spliceostatin, is a potent inhibitor of eukaryotic RNA splicing by virtue of its affinity to the SF3b subunit of the U2 snRNA subcomplex of the spliceosome [16, 17]. As the ADC field is actively looking for cytotoxic payloads beyond the microtubule inhibitor class [1, 18], we recently introduced thailanstatin as a potent payload for lysine conjugated ADCs [19]. In this report, we show that there is a high dependence of the potency of thailanstatin ADCs with the site of conjugation. The identification of these sites on the antibody was greatly facilitated by the development of a new methodology for the loading of multiple payloads onto a single cysteine site using a peptidic linker. The site-specific thailanstatin ADCs were shown to be very efficacious in an *in vivo* gastric cancer tumor xenograft model thus demonstrating the suitability of this novel class of payload for consideration in next-generation site-specific ADC programs.

## Results

### Hinge cysteine conjugates of thailanstatin analogs show poor *in vivo* efficacy in Her2 positive Xenograft mice models of cancer

**Fig 1:**
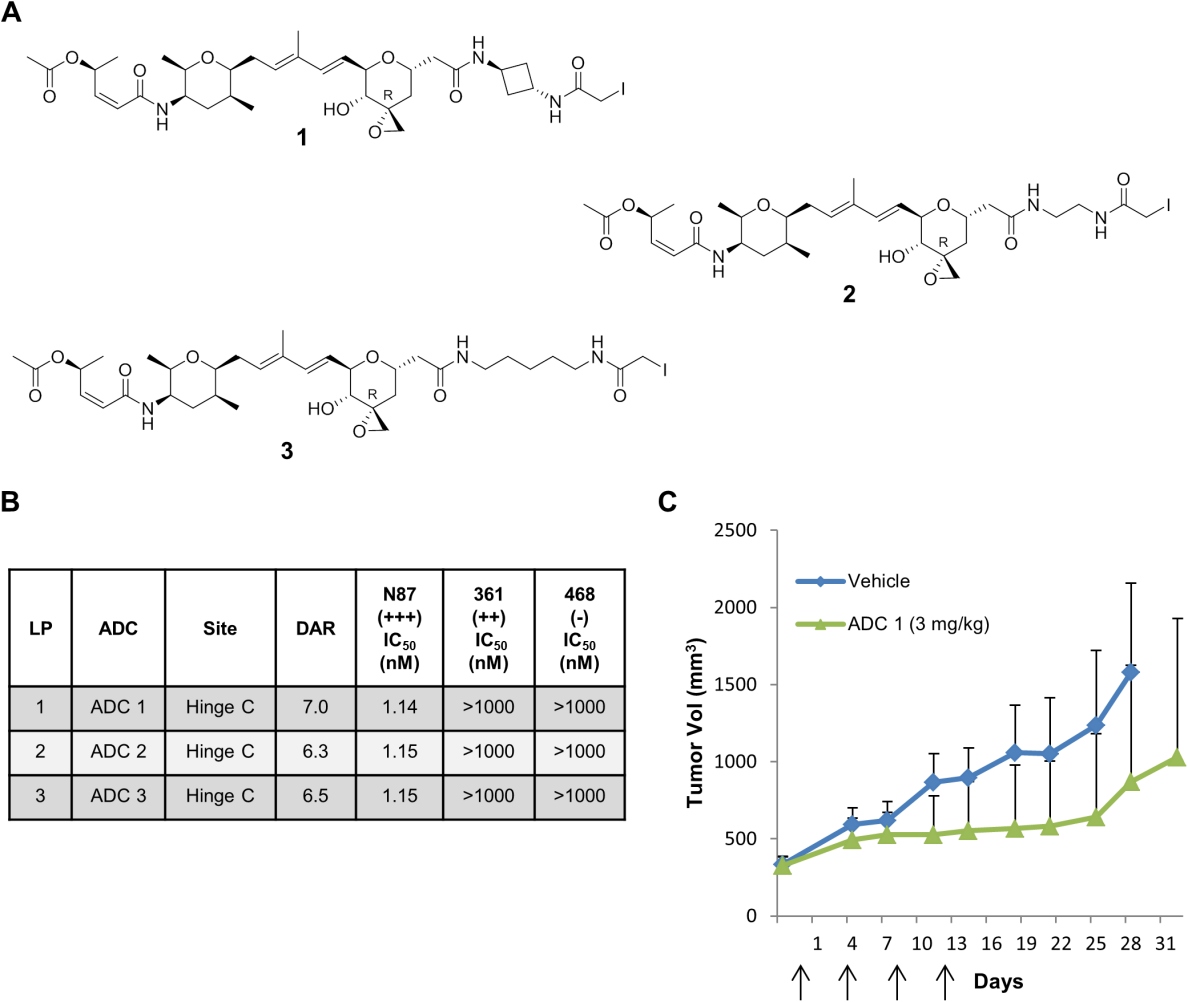
Potency of hinge-cysteine thailanstatin trastuzumab ADC. (A) Structure of iodoacetamide derivatized non-cleavable thailanstatin linker-payloads (LPs). (B) *In vitro* cytotoxicity of hinge-cysteine thailanstatin trastuzumab ADCs against cancer cell lines expressing various levels of Her2, reported in half-maximal inhibitory concentration (IC_50_) values of conjugated payload in nM. Data are the mean of multiple experiments. (C) *In vivo* efficacy of hinge-cysteine thailanstatin trastuzumab **ADC1** in an N87 gastric cancer xenograft model dosed at 3 mg/kg (q4d x 4). Arrows indicate the day(s) on which intravenous dosing was carried out. DAR = Drug Antibody Ratio; 361 = MDA-MB-361-DYT2; 468 = MDA-MB-468.

Three iodoacetamide thailanstatin linker-payloads with varying linker lengths (1–3; Fig 1A) [20] were prepared and conjugated to the hinge cysteines of anti-Her2 trastuzumab antibody following partial reduction of the interchain disulfides bonds using tris(2-carboxyethyl)phosphine (TCEP). The average drug-antibody ratio (DAR) of these trastuzumab ADCs as measured by liquid chromatography-mass spectrometry (LC-MS) ranged from 6.3 to 7.0. The cytotoxicity and selectivity of these ADCs (**ADC1-3**) were assessed against several cancer cell lines with varying levels of Her2 expression (Fig 1B). Similar to our earlier published results [19], all the hinge cysteine conjugates of thailanstatin displayed potent activity (IC_50_~1 nM) against the high Her2 (>600,000 receptors/cell) expressing N87 gastric cancer cell line but were not potent against moderate antigen expressing MDA-MB-361-DYT2 (361) breast cancer cell line (~150,000 receptors/cell). As expected, all ADCs were inactive against a non-antigen expressing cell lines, MDA-MB-468 (468). Because of their potency in the N87 cell line, one of the ADCs (**ADC1**) was chosen to be evaluated for efficacy in an N87 gastric cancer xenograft model in nude mice. Eight randomized animals were dosed intravenously at 3mg/kg on days 0, 4, 8 and 12 (q4d x 4) when the tumor size reached approximately 350 mm^3^. In contrast to the excellent potency previously reported for DAR4 lysine thialanstatin conjugates [19], only a modest reduction in tumor growth rate was observed in comparison to a vehicle control (Fig 1C).

### Single cysteine site-specific conjugation of thailanstain linker-payload does not improve potency against moderate Her2 expressing cancer cells

The cytotoxicity of ADCs that were generated by site-specific conjugation of the thialanstatin LP **2** to a variety of antibodies that incorporated engineered cysteine residues (kappa light chain K183C; heavy chain A114C, K334C, Q347C, E388C, K392C, L443C) were evaluated (Table 1). Auristatin and tubulysin conjugates of these site-mutant antibodies have been described previously [21, 22]. Unpaired cysteines are typically isolated as capped species, wherein the cysteine residue is blocked with glutathione or other small molecule thiol. The eight site-specific ADCs (Table 1, **ADC4-11**) were uncapped by first reducing the engineered cysteines and the interchain disulfides using excess TCEP followed by the re-oxidation of the hinge cysteines using dehydroascorbic acid (DHA) and treatment with excess linker-payload **2**. After workup and purification, the DAR of the resulting site-specific ADCs ranged from 1.7–2.0. Similar to the hinge cysteine conjugates, the site-specific single cysteine based ADCs showed excellent potency against high Her2 expressing N87 cell lines (0.4 to 9 nM, Table 1), but were not potent against the moderate Her2 expressing 361 cancer cell line (IC_50_ >800 nM). The relative exposure of the conjugated payload to bulk solvent was assessed by hydrophobic interaction chromatography, as previously described [12]. The hydrophobic retention time (HIC) relative retention times (HIC RRT) for each ADC was calculated by dividing the retention time of the ADC by that of unconjugated trastuzumab antibody.

**Table 1:** In vitro cytotoxicity of single-cysteine mutant thailanstatin trastuzumab ADCs against various levels of Her2 expressing cancer cell lines, reported in Mean IC_50_ values of conjugated payload in nM.

LP	ADC	Site	HIC RRT	DAR	N87 (+++) IC_50_ (nM)	361 (++) IC_50_ (nM)	468/HT29* (-) IC_50_ (nM)
2	ADC 4	114	1.14	1.9	0.46	>1000	>1000
2	ADC 5	k183	1.12	2	0.98	>1000	>1000
2	ADC 6	421	1.33	2	4.23	>1000	>1000
2	ADC 7	443	1.36	2	0.83	>1000	>1000
2	ADC 8	392	1.08	1.7	0.56	>805	>805*
2	ADC 9	334	1.08	2	0.65	>805	>805
2	ADC 10	347	1.23	2	9.18	>805	>805*
2	ADC 11	388	1.27	2	0.96	>805	>805

Data are the mean of multiple experiments. k = kappa light chain; all other mutations are on heavy chain.

### Development of a multi-payload carrying peptidic linker to increase the DAR of single-cysteine antibody-drug-conjugate

**Fig 2:**
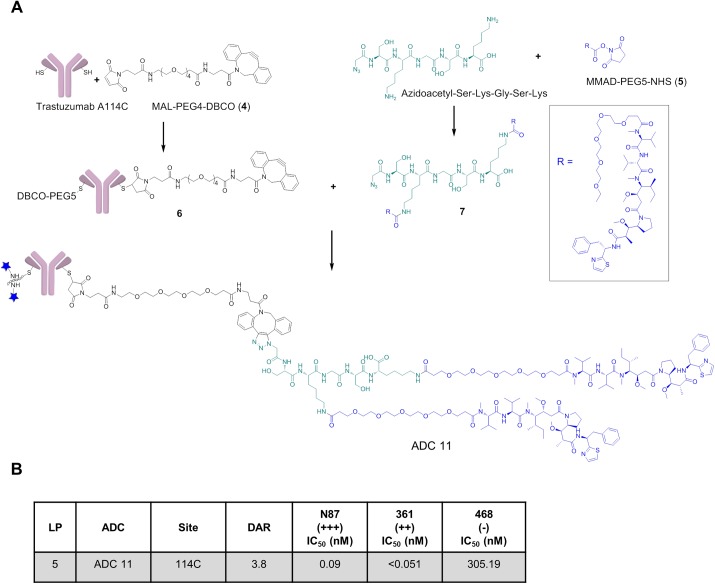
Generation of site-specific multiple-payload carrying peptidic linker (MPP) ADC delivering MMAD. (A) Schematic showing generation of a double-MMAD carrying peptidic linker ADC generated on trastuzumab A114C. (B) *In vitro* cytotoxicity of peptidic linked MMAD trastuzumab A114C ADC against various levels of Her2 expressing cancer cell lines, reported in Mean IC_50_ values of conjugated payload in nM. Data are the mean of multiple experiments. MAL = malemide; DBCO = Dibenzocyclooctyne.

As we have previously shown that the potency of lysine thailanstain ADCs [19] are highly dependent upon DAR, we wanted to evaluate if increasing the DAR at the engineered cysteine sites also improved the cytotoxicity of the thailanstain ADCs toward the 361 cell line. We envisioned increasing loading to an engineered cysteine by utilizing click chemistry to conjugate a small, multiple-payload carrying peptide in a two-step conjugation method. To validate this methodology for ADC generation, we employed a widely utilized ADC payload, auristatin MMAD. A multi-payload carrying peptidic linker (MPP) was generated *in situ* and subsequently conjugated to a model antibody (Fig 2A) by click chemistry. The conjugation was performed using a single engineered cysteine antibody (trastuzumab A114C) preprogrammed with a strained alkyne, malemide-PEG4-Dibenzocyclooctyne (DBCO). (Fig 2A, **4**) Concurrently, we generated a two-payload carrying azido peptide by conjugating the lysines of the N-terminal azidoacetyl substituted peptide (SKGSK) with an N-hydroxysuccinimide (NHS) ester of PEG5-MMAD (Fig 2A, **5**). A small, fairly neutral 5-mer peptide with two lysines separated by glycine-serine was chosen to reduce any potential instability. The excess NHS ester was quenched with ethanolamine and the resulting double-payload carrying peptide linker (**7**) was then conjugated to the DBCO-preprogrammed antibody (**6**) via an *in situ* copper free click reaction thus yielding a site-specific ADC with a DAR of 3.8 (Fig 2A, **ADC11**). This ADC was purified using size-exclusion chromatography (SEC) and characterized by LC-MS and analytical SEC. The cytotoxicity of **ADC11** was measured against cancer cell lines with various levels of Her2 expression and the ADC was found to be extremely potent against Her2 expressing N87 (IC_50_ = 0.49 nM) and 361 (IC_50_ = 0.39 nM) cell lines and exhibited approximately 1000 fold selectivity against the non-Her2 expressing 468 cell line. By comparison, a DAR4 lysine conjugate of **5** (not shown) exhibited an IC_50_ of 0.95 nM against N87 cells and 0.16 nM against 361 cells.

### *In vitro* and *in vivo* potency of ADCs generated using the double-thailanstatin carrying peptidic linker onto single cysteine mutants of trastuzumab

**Fig 3:**
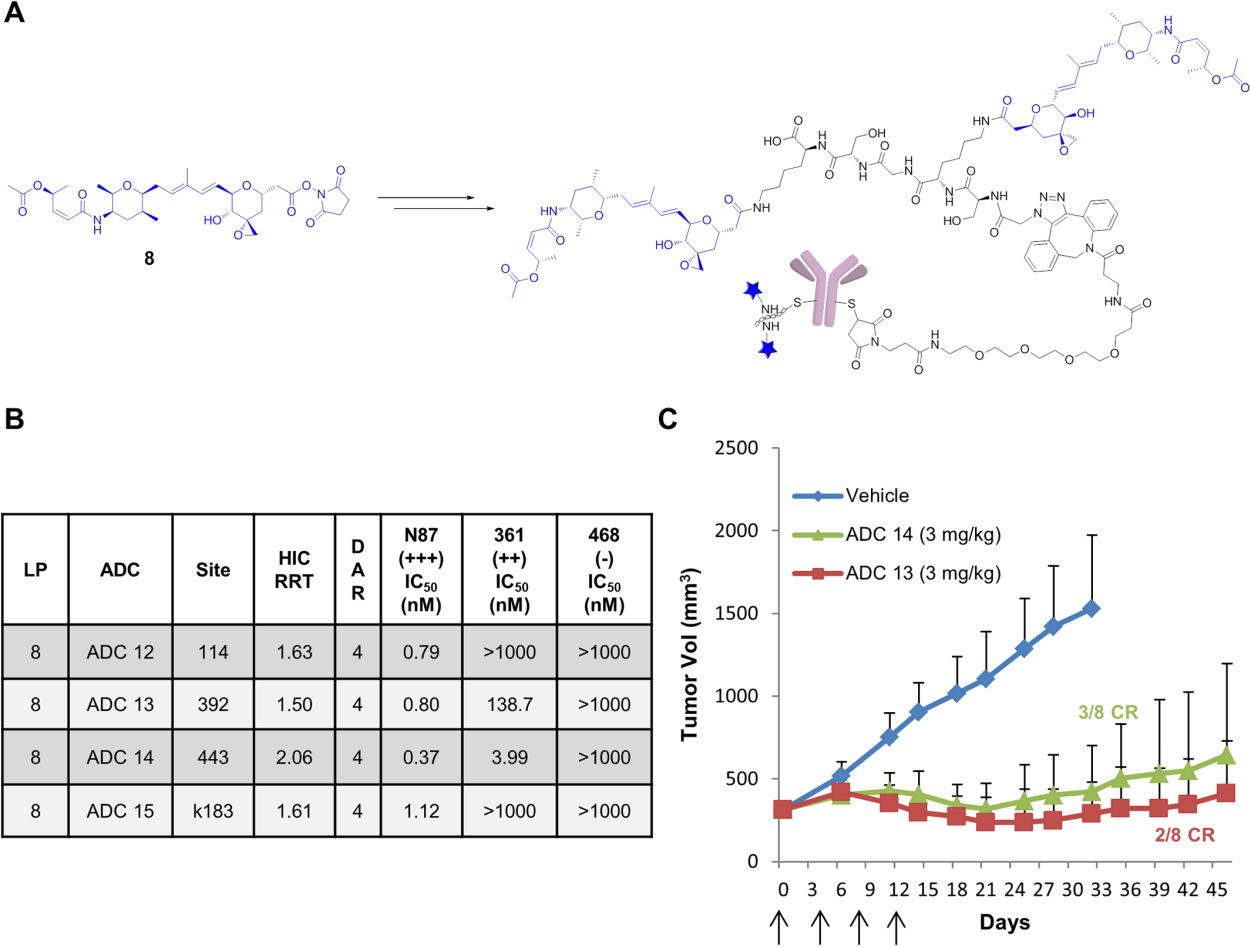
Site-specific thailanstatin MPP ADCs. (A) Preparation of thailanstatin MPP ADC from thailanstatin NHS ester (**8**). (B) *In vitro* cytotoxicity of site-specific thailanstatin MPP trastuzumab ADCs against various levels of Her2 expressing cancer cell lines, reported in Mean IC_50_ values of conjugated payload in nM. Data are the mean of multiple experiments. (C) *In vivo* efficacy of thailanstatin MPP trastuzumab ADCs (**ADC 13** and **ADC 14**) in N87 gastric cancer xenograft model dosed at 3 mg/kg (q4d x 4). Arrows indicate the day(s) on which intravenous dosing was carried out.

Having demonstrated the ability to load an auristatin payload using the MPP chemistry, the N-terminal azidoacetyl SKGSK peptide was conjugated to the NHS ester of thailanstatin A (Fig 3A, **8**) to yield a double thailanstatin conjugated azidopeptide. This dual-loaded peptide was then conjugated to four different DBCO preprogrammed single cysteine mutant trastuzumab antibodies using copper free click conjugation (Fig 3A). This methodology enabled us to increase the DAR of single cysteine thailanstatin ADCs from 2 to 4 in order to explore the site-dependent activity of the thailanstatin payload. The four engineered cysteine sites were selected based on their different solvent exposures as assessed by their HIC RRT. A high HIC RRT suggests that the payload has relatively higher exposure to bulk solvent while a low HIC RRT suggests that the payload is more hidden in the antibody tertiary structure [12]. As such, based on the HIC retention times of single cysteine thailanstatin trastuzumab ADC (Table 1) one highly exposed site was selected (443C, HIC RRT = 1.36) and three moderate to low exposure sites were selected (392C, HIC RRT = 1.08; 114C, HIC RRT = 1.14; and kappa-183C, HIC RRT = 1.12). As observed with the DAR2 ADCs, all the MPP ADCs exhibited excellent potency against the N87 cell lines and high IC_50_ in the non-Her2 expressing 468 cell line (Fig 3B). Surprisingly, however, three of the ADCs showed improved *in vitro* potency against the moderate Her2 expressing 361 cell lines. In particular, **ADC14** (conjugated at the 443C site) exhibited low nM activity against the 361 cell line followed by **ADC13** (392C) > **ADC12** (114C) > **ADC15** (k183C).

Encouraged by these results, we evaluated the MPP ADCs generated on sites 443 and 392 (**ADC14** and **ADC13**, respectively) in the nude mouse N87 xenograft tumor model. The mice were injected with ADCs intravenously at two different doses of 1.5 mg/kg and 3 mg/kg as described previously. While at the 1.5 mg/kg dose the ADCs were only marginally efficacious (S1A Fig), significant tumor regression was observed at the 3mg/kg dose for both of the MPP ADCs (Fig 3C). Complete regression (CR) was observed in three out of eight mice that were injected with 443C MPP ADC and in one out of eight mice that were injected with the 392C MMP ADC. (S1B and S1C Fig).

### Improved *in vitro* and *in vivo* efficacy of selected double-cysteine mutant trastuzumab ADC

**Fig 4:**
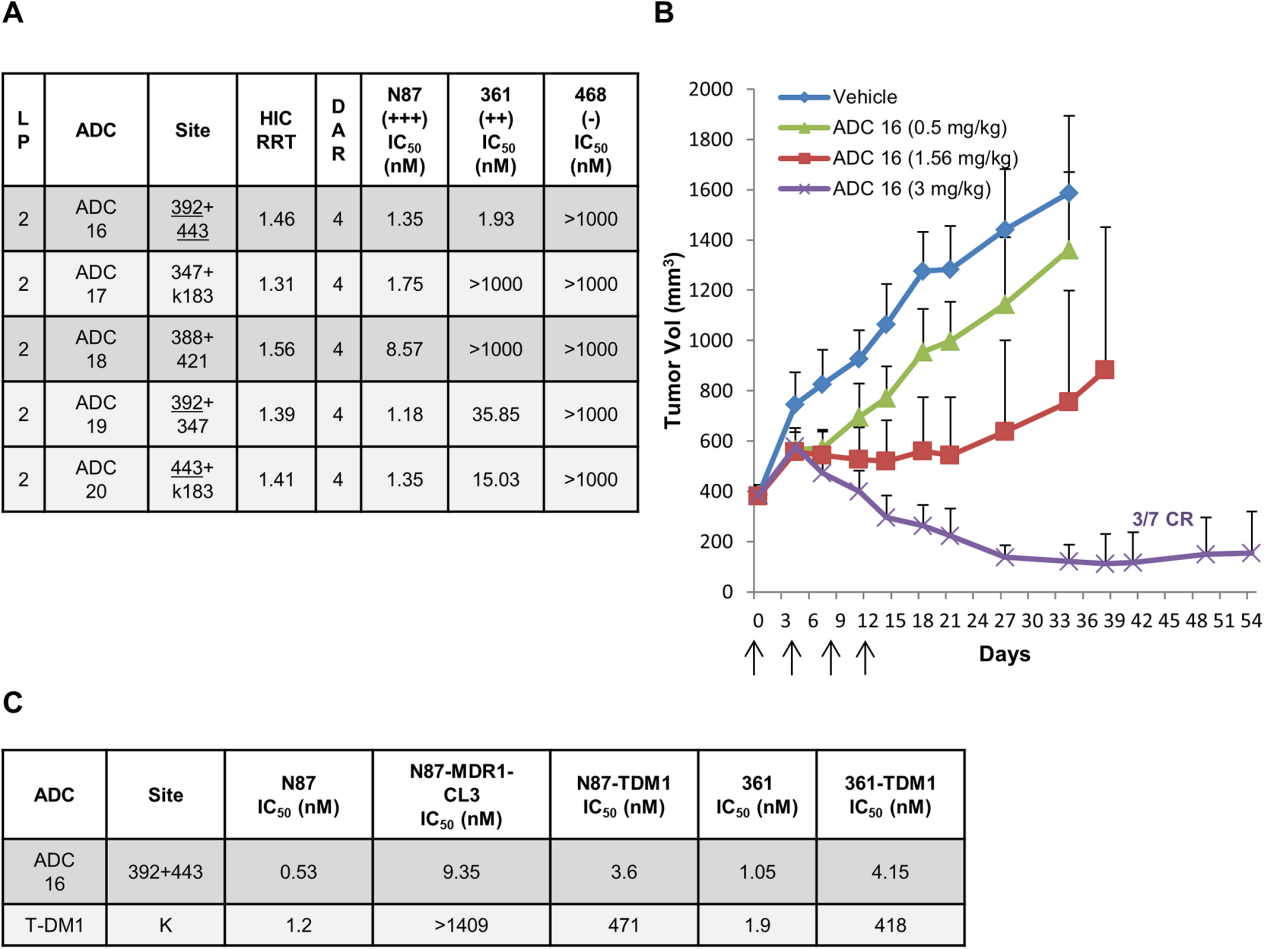
Double-cysteine mutant thailanstatin trastuzumab ADCs. (A) *In vitro* cytotoxicity of double-cysteine mutant thailanstatin trastuzumab ADCs against various levels of Her2 expressing cancer cell lines, reported in Mean IC_50_ values of conjugated payload in nM. Data are the mean of multiple experiments. (B) *In vivo* efficacy of double-cysteine mutant thailanstatin trastuzumab **ADC16** in N87 gastric cancer xenograft model dosed at 0.5, 1.56 and 3 mg/kg (q4d x 4). (C) *In vitro* cytotoxicity of double-cysteine mutant thailanstatin trastuzumab **ADC16** against T-DM1 resistant N87 (N87-TDM1) and 361 (361-TDM1) as well as MDR1 overexpressing N87 (N87-MDR1-CL3) cancer cell lines, reported in IC_50_ values of conjugated payload in nM.

Based on the improved efficacy of MPP ADCs generated on the 392 and 443 sites, we wanted to explore the combination of these sites to further improve the potency of thailanstatin trastuzumab ADCs. To this end, we engineered a double mutant, trastuzumab-392+443 and generated the corresponding DAR4 site-specific **ADC16** conjugated with iodoacetamide thailanstain linker-payloads (**2**). Gratifyingly, this double mutant showed much improved activity against moderate Her2 expressing 361 cell lines and maintained potent cytotoxicity towards the N87 cell lines in the *in vitro* cytotoxicity assay. To assess if the activity against the moderate antigen expressing cell lines is not just a characteristic of higher loaded site-specific ADC, we evaluated the cytotoxicity of four more ADCs (**ADC17-20**) generated by other combinations of engineered cysteine sites, 347+k183, 388+421, 347+392 and 443+k183. Two of the ADCs that were generated on antibodies where at least one site of conjugation was either 443 or 392 (**ADC19** and **ADC20**) exhibited improved cytotoxicity against the 361 cell lines, as compared to the other two ADCs that were not conjugated at these preferred sites (**ADC17** and **ADC18**) (Fig 4A).

We further evaluated the 392+443 double mutant (**ADC16**) in an N87 tumor xenograft bearing nude mouse study as described above. Three doses (0.5, 1.5, and 3 mg/kg) were administered (IV) and a dose dependent tumor regression by this site-specific ADC was observed (Fig 4B). Substantial regression of tumor was observed at the 3 mg/kg dose resulting in complete regression for three out of seven mice by day 41 (S2A Fig). To further assess the therapeutic potential of this site-specific thailanstatin ADC, we evaluated its *in vitro* cytotoxicity against a T-DM1 resistant [23, 24] and MDR1 overexpressing [19] cancer cell lines and found it to be 20–100 times more potent than T-DM1 (Fig 4C).

## Discussion

Despite the success of ADCs like ADCETRIS^®^ and KADCYLA^®^, there are a number of microtubule inhibitor (MTI) ADCs which continue to suffer from poor therapeutic efficacy in the clinic [25]. One approach being explored by researchers to improve efficacy is to identify highly potent non-MTI payloads which are effective against slow-growing tumor cells and can overcome MDR mediated chemotherapeutic resistance [26, 27]. In fact, next generation DNA damaging agents are already being investigated in clinical studies and several other payloads with alternative mechanism of action are also being explored preclinically [1, 18]. We recently introduced thailanstatin, a potent cytotoxic with a novel mechanism of action, as a payload for antibody drug conjugates. Thailanstatin A is a natural product initially isolated from *Thailandensis burkholderia* MSMB43 that exerts its activity by binding to the SF3b subunit of the U2 snRNA subcomplex of the spliceosome [16, 28, 29]. We have reported a genetic modification of *T*. *burkholderia* that allows for gram-scale production of thailanstatin A [30]. The mechanism of cytotoxicity of this payload is through inhibition of the eukaryotic mRNA splicing pathway which results in low to sub-nanomolar IC_50_ values against multiple cancer cell lines. Overexpression of MYC, an aggressive oncogene, is one of the most common drivers of human cancer and MYC dependent cell lines have remained resistant to therapeutics. Recently, Westbrook et al. reported that *in vivo* inhibition of the spliceosome impairs tumorigencity of MYC-dependent breast cancers and their metastatic potential [31]. Thus, our interest in this payload class extends beyond its excellent potency against quiescent and MDR expressing cancer cell lines [19, 32] due to its potential therapeutic benefits against the MYC-driven cancers.

We have previously attempted to generate a potent thailanstatin ADC via conjugating a haloacetamide thailanstatin linker-payload to the hinge cysteines of trastuzumab. Although this ADC displayed excellent potency against high Her2 expressing cancer cell lines, cytotoxicity against the moderate antigen expressing 361 cell line was lacking [19]. This was surprising considering that as a small molecule, this class of payload has potency [33] similar to that of many MTIs such as MMAE, which are very efficacious ADC payloads. In order to further probe this puzzling result and improve activity against moderate antigen expressing cell lines, we explored additional linkers of varying length and rotational restriction (Fig 1A). Consistent with our prior report, the IC_50_ values of the resulting hinge-cysteine ADCs were very high against the 361 cell lines in spite of their high DARs (6.3 to 7.0). Despite lower potency against the moderate Her2 expressing 361 cell lines, we wanted to evaluate these ADCs *in vivo* in an N87 gastric cancer xenograft model due to their potent *in vitro* activity against the N87 cell line. Therefore, one of the thailanstatin ADCs (**ADC1**) which displayed excellent *in vitro* potency against a high Her2 expressing N87 cell line was selected for *in vivo* evaluation. Dosed at 3 mg/kg (qdx4), this ADC exhibited only marginal activity in an N87 gastric cancer xenograft model compared to the vehicle control (Fig 1C). This efficacy was much lower than that observed for lower loaded lysine conjugates of trastuzumab to thailanstatin A, which exhibited significant tumor regression in this model [19]. These lysine conjugates also showed much improved cytotoxicity against the moderate antigen expressing 361 cell line. The lower *in vivo* efficacy of the hinge cysteine ADCs correlated well with their poor *in vitro* potency against the moderate antigen expressing 361 cell line. We hypothesized that the superior potency of the lysine conjugate could perhaps be attributed to the heterogeneity of the ADC, whereby some conjugation sites within the mixture may impart improved lysosomal processing and potency to the final ADC. Therefore, we designed a set of site-specific conjugates in order to identify sites that might result in improved potency of thailanstatin ADC towards the 361 cell line. In addition, a site specific conjugate of thailanstatin may provide benefits such as improved manufacturability, bioanalytical characterization, pharmacokinetics and safety as compared to its lysine counterpart [9].

The retention time of an ADC by hydrophobic interaction chromatography is partially dictated by the accessibility of the conjugated payload to interact with the stationary phase, which in turn, is dependent upon how exposed the site is to bulk solvent. Our recently reported work suggests that there is a strong correlation of HIC retention time of site-specific ADCs with metabolic stability [12]. Other reports have suggested that lower HIC retention correlates with decreased antibody clearance and improved exposure [34]. To assess if the same trends hold true for thailanstatin ADCs, linker-payload **2** was conjugated to several different conjugation sites that resulted in ADCs with varying levels of hydrophobicity as measured by their HIC retention times (Table 1). These site-specific ADCs (with DARs of about two) were found to be potent against the high Her2 expressing N87 cell line (IC_50_’s of 0.47 to 9.2 nM) and were selective against the non-antigen expressing cell lines (IC_50_’s of >1000 nM). However, none of the ADCs were very potent against the moderate Her2 expressing 361 cell line, exhibiting IC_50_’s of > 1000 nM (Table 1). While the average DAR of the previously described active lysine based thailanstatin ADC was two, this ADC was a mixture of conjugates with high and low DAR. Thus, we postulated that the activity of this conjugate was derived both from the optimal site of conjugation and possessing a higher DAR.

In order to address this hypothesis, we developed a novel method to generate a site-specific ADC with a DAR of 4 by conjugating a double-payload loaded peptidic-linker generated *in situ* to a single site (Fig 2A). We wanted to first validate this methodology and verify if a viable ADC could be produced which showed good potency in antigen positive cells and good selectivity. Micro-tubule inhibiting auristatins such as MMAD are one of the most widely used ADC payloads. Therefore, as a proof of concept, the NHS ester of MMAD (**5**), was conjugated to the two lysines of a peptide (SKGSK) derivatized with an azidoacetyl group at the N-terminus. The resulting double-MMAD loaded azido-peptide (**7**) was conjugated *in situ* to trastuzumab A114C that had been pre-loaded with a maleimide DBCO (**6**). This four loaded site-specific MPP ADC (Fig 2A, **ADC11**) showed potent cytotoxicity against the antigen expressing cell lines and was selective against non-antigen expressing cell lines (Fig 2B), validating this methodology to generate potent and selective ADCs.

Having established a methodology that allowed us to explore higher DAR conjugates using a single site of conjugation, we proceeded to prepare the corresponding thailanstatin ADCs (Fig 3) using four different cysteine site mutants: 443C being the most solvent exposed and 114C, kappa-183C and 392C with moderate to low solvent exposure based on their HIC RRT values [12], After confirming a DAR of 4 by LC-MS for each of the thailanstatin MMP ADCs, the conjugates were assessed for their cytotoxicity against various cancer cell lines. Interestingly, we observed site-dependent improved potency against the moderate Her2 expressing 361 cell line by some of these MPP ADCs and unlike our previous experiences [12, 35], the activity of these site mutants did not correlate with the hydrophobicity of the ADC. Both the highest and lowest hydrophobic conjugates displayed improved potency with an IC_50_ of 5.8 nM for the 443C site and 195 nM for the 392C site, whereas the MPP ADCs generated on the moderately hydrophobic sites were less potent (Fig 3B). Interestingly, the considerable improvement in 361 activity of **ADC20** was accompanied by no significant improvement in potency against the high-Her2 expressing cell line N87. Previously, we have shown that the lysine ADC of the des-acetyl thailanstatin metabolite of thailanstatin A displayed similar potency to the parent ADC [19]. An identical ranking of site-dependent activity of 443>392>114>k183 was observed with MPP ADCs generated with NHS ester of equally potent des-acetyl thailanstatin metabolite (data not shown). Encouraged by their improved 361 potency, the 443 and 392 MPP ADCs were evaluated for their *in vivo* efficacy against the N87 gastric cancer tumor xenograft model in nude mice at 1.5 mg/kg and 3mg/kg. Significant tumor regression was observed at 3mg/kg for both the MPP ADCs and, more importantly, three out of eight mice were cured by the 443 MPP ADC and one out of eight by the 392 MPP ADC (Fig 3C; S1B and S1C Fig).

A mechanistic rationale for the improvement in the activity of the ADC conjugated to these specific sites is not known at this time and further studies are underway to understand this phenomenon. The dependence on position appears to be most important in cancer cells expressing low to moderate levels of antigen, which is a tumor population with a high barrier for targeted therapies. Nevertheless, these results caution against screening novel ADC payloads on just a few “stable” sites as each class of payloads might interact unpredictably with its immediate protein environment, resulting in different potencies. It is conceivable that the vicinal chemical environment around the site of conjugation could potentially impart improved potency by shielding the payload against metabolic enzymes in the lysosomal environment or by facilitating faster payload release. Regardless of the underlying mechanism, these results indicate that the efficacy of certain site-specific conjugates is not solely dictated by the improved stability due to the increased steric or low solvent exposure as measured by HIC retention times.

Based on the improved potency and efficacy of the 443 and 392 MPP ADCs, we hypothesized that a double-cysteine ADC generated by conjugating thailanstatin iodoacetamide payload directly to the 443 and 392 sites might show a similar level of improvement in efficacy. Therefore, a double cysteine mutant of trastuzmab 443 + 392 was generated and linker-payload **2** was conjugated to achieve a site-specific ADC with a DAR of 4 (**ADC16**). *In vitro* cytotoxicity against the moderate antigen expressing 361 cell line indeed displayed excellent potency with much improved IC_*50*_ values while maintaining potency against the N87 cell line and selectivity against the non-expressing 468 cell lines (Fig 4A). This is in contrast to the same linker-payload conjugated to the hinge cysteines, with a much higher DAR, which exhibited very poor potency against the 361 cell line (Fig 1, **ADC1**). This data clearly suggests that site of conjugation can play a critical role in the enhancement of thailanstatin ADC cytotoxicity.

To further probe this hypothesis, four more double cysteine mutants were generated, two of which contained one of the preferred sites, 392 or 443 (**ADC19** and **ADC20**, Fig 4A). Similar to the 443+392 ADC (**ADC16**), a loading of four linker-payloads was obtained for these double-cysteine mutants. The conjugates derived from both trastuzumab (T)-347C+k183C (**ADC17**) and T- 388C+421C (**ADC18**) were not very potent against the 361 cell lines, whereas T-347C+392C (**ADC19**) and T-443C+k183C (**ADC20**), both of which carry one of the preferred sites, exhibited slightly improved potency against the 361 cell line (Fig 4A). However, the cytotoxicity of 443+392 ADC was still the best among all the screened double–cysteine based thailanastain ADCs. These results further indicate that the thailanstatin potency is highly site-dependent and that the increase in potency is derived not just from the increase in DAR but also from the attachment to certain privileged sites, such as 443C and 392C.

We further evaluated the best double-cysteine thailanstatin trastuzumab 392+443 **ADC16** for its *in vivo* efficacy against the N87 gastric cancer tumor xenograft model. At 3 mg/kg this ADC showed excellent tumor regression and three out seven mice were completely cured by day 41, while no significant change in the body weight was observed (Fig 4B, S2A and S2B Fig). This is a very interesting result considering that a higher loaded hinge cysteine conjugate (**ADC1**, Fig 1C) was only marginally active at 3 mg/kg while the analogous site-specific **ADC16** resulted in effective tumor regression at the same dose. **ADC16** was tolerated at up to 10 mg/kg in rats and no significant body weight change or overt toxicity was observed (data not shown). This ADC is also cytotoxic against cell lines that are resistant to the clinically approved ADC, T-DM1 [23, 24] (Fig 4C), furthering its attribute as a payload of choice for the next generation of ADCs advancing in the clinic. MDR1 overexpression in tumor cell is a key mechanism of chemotherapy resistance and T-DM1 has been shown to be about 1000 fold less sensitive to these MDR1 overexpressing Her2 positive cell lines [36]. In these cell lines, **ADC16** was over 100 times more potent than T-DM1 (Fig 4C), which is indicative of its potential against chemotherapy-resistant MDR1 expressing tumor.

## Conclusion

In this study we describe the identification of a highly efficacious, site-specific thailanstatin trastuzumab ADC with a unique cytotoxic mechanism of action. Screening for an effective site of conjugation on the antibody was challenging as it defied our prior experience with tubulysin and auristatin payloads, where site of conjugation did not significantly impact the *in vitro* cytotoxicity of the ADC. These results emphasize the importance of screening novel classes of ADC payloads on multiple sites rather than simply at a single “preferred” site. We show that potency of the thailanstatin ADC is the result both of high DAR as well of the specific sites of conjugation. The development of a novel multi-payload carrying peptidic (MPP) linker ADC allowed us to screen for these preferred conjugation sites by increasing the DAR on a single site of attachment. MPP ADCs and the double-cysteine mutant ADC generated on these sites showed substantially improved tumor regression in comparison to the approved drug T-DM1 (S3 Fig) [19] with the additional advantage of utilizing a drug with a distinct MOA which may be advantageous against the T-DM1 resistant and MDR positive tumors.

## Method

### Preparation of hinge-cysteine ADC

Commercially available trastuzumab (Genentech Inc) was dialyzed into Dulbecco’s Phosphate Buffered Saline (DPBS, Lonza). The dialyzed antibody is reduced with addition 5 equiv of tris(2-carboxyethyl)phosphine hydrochloride (TCEP, 5 mM in distilled water) and diluted to 15 mg/mL final antibody concentration using DPBS, 5 mM 2, 2', 2", 2‴-(ethane-1, 2-diyldinitrilo)tetraacetic acid (EDTA), pH 7. 0. The reaction is incubated at 37°C for 1.5 h and then cooled to room temperature. Conjugation was performed in 50 mM borate buffer, pH 8.5 by addition of 11 equiv of iodoacetamide thailanstatin payload (1–3) [20] that was dissolved as a 10 mM solution in dimethylacetamide (DMA). DMA was added to achieve a 10% (v/v) total organic level. The conjugation was performed at 10 mg/mL for 16 h at room temperature. The ADC was purified and characterized as described earlier [21]. Briefly, the reaction mixture was buffer exchanged into DPBS (pH7. 4) using GE Healthcare Sephadex G-25 M buffer exchange columns per manufacturer's instructions. Crude material was purified by size exclusion chromatography (SEC) using a GE AKTA Explorer system with a GE Superdex 200 column and DPBS (pH 7. 4) eluent. The pooled monomer fractions were concentrated using 50kDa molecular weight cut-off (MWCO) AmiconUltra-15 centrifugal unit (Millipore, Billerica, MA), if required. The resulting ADCs were filtered through sterile 0.22 μm Ultrafree-MC Centrifugal Filter Units (Millipore, Billerica, MA) and concentration was measured spectrophotometrically. The ADCs were further characterized via size exclusion chromatography (SEC) for purity and liquid chromatography electrospray ionization tandem mass spectrometry (LC-ESI MS) to calculate drug antibody ratio (loading) and confirm mass shift.

### Preparation of site-specific cysteine ADC

Site-directed mutagenesis, antibody expression, and purification were performed as previously described [22]. The antibodies used in the conjugation studies were dialyzed into Dulbecco’s Phosphate-Buffered Saline (DPBS, Lonza). Conjugation of the Iodoacetamide-functionalized linker-payloads to the antibodies carrying engineered cysteines was conducted as described earlier[21] with the following changes. The antibody was diluted to 10 mg/mL with DPBS containing 5 mM 2,2′,2″,2‴-(ethane-1,2-diyldinitrilo) tetraacetic acid (EDTA). The resulting antibody was treated with 50−100 equiv of tris (2-carboxyethyl) phosphine hydrochloride (TCEP, 5 mM in distilled water) and incubated at room temperature for 1−2 h. The reaction mixture was then buffer exchanged into DPBS containing 5 mM EDTA using GE Healthcare Sephadex G-25 M buffer exchange columns per the manufacturer’s instructions. To re-form the interchain disulfide bonds, the antibody was then reoxidized using 40 equiv of dehydroascorbic acid (DHA) overnight at 4°C. The reaction mixture was then buffer exchanged into DPBS containing 5 mM EDTA (pH 7.0). Conjugation was performed in 50 mM borate buffer, pH 8.5 by addition of 11 equiv of **2** that was dissolved as a 10 mM solution in DMA. The conjugation was performed at 10 mg/mL for 16 h at room temperature. The ADC was purified and characterized as described earlier [21]. Briefly, the reaction mixture was buffer exchanged into DPBS (pH7. 4) using GE Healthcare Sephadex G-25 M buffer exchange columns per manufacturer's instructions. The resulting ADC was then purified as mentioned above and subsequently filter-sterilized and characterized by analytical SEC, HIC, LC/ESI MS to calculate the DAR and mass shift.

### Cell lines and cytotoxicity assay

Cell lines and Cytotoxicity Assay: N87 (gastric cancer) and MDA-MB-468 (breast cancer) cell lines were obtained from ATCC (Manassas, VA); MDA-MB-361-DYT2 (breast cancer) cells were generously provided by Dr. Dajun Yang at Georgetown University. TDM1-resistant MDA-MB-361-DYT1-TM and N87-TM cells were generated as described previously [23, 24]. N87-MDR1 cell were generated as described previously[21]. Cells were maintained in RPMI (N87), DMEM (468), or MEM (361) media supplemented with 10% fetal bovine serum (FBS), 1% L-glutamine, 1% sodium pyruvate. Cytotoxicity assessment was determined as reported previously[23]. Briefly, cells were treated with ADCs or compounds for 4 days, then cell viability assessed with CellTiter® 96 AQueous One MTS Solution (Promega, Madison, WI). IC_50_ values were calculated using a four-parameter logistic model with XLfit (IDBS, Bridgewater, NJ) and are the arithmetic mean of multiple determinations.

### N87 *in vivo* efficacy model

All animal studies were approved by the Pfizer Institutional Animal Care and Use Committee according to established guidelines. Female athymic nu/nu (Nude, Stock No: 002019 mice obtained from The Jackson Laboratory (Farmington, CT) were injected subcutaneously in the flank with suspensions of 1 X 10^6^ of N87 cells respectively in 50% Matrigel (BD Biosciences, Franklin Lakes, NJ). Mice were randomized into study groups when tumors reached approximately 150 mm^3^ to 300 mm^3^. Either phosphate buffered saline (PBS, Gibco, Cat#14190–144, as vehicle), trastuzumab-thailanstatin ADCs with different DAR, or PT-DM1 at different doses were administered intravenously starting on day 0 for a total of four doses, 4 days apart (Q4d x4). Tumors were measured at least weekly with a calibrator (Mitutoyo, Aurora, Illinois) and the tumor mass was calculated as volume = (width X width X length)/2.

### Preparation of multiple-payload carrying peptidic linker (MPP) ADC

Conjugation of cysteine-mutant antibody with Malemide-PEG4-DBCO (**4**; Sigma-Aldrich, catalog no. 760676) was performed as described earlier.[21] Briefly, following the reduction and re-oxidation of the antibody as mentioned above, 5 equiv of 4 (10 mM in DMA) was added to 10mg/mL of the prepped antibody in DPBS, 5 mM EDTA. The reaction was incubated for 2h at room temperature and purified as described above to yield 6. The DBCO functionalized antibody was then concentrated to over 10 mg/mL concentration using 50kDa MWCO AmiconUltra-15 centrifugal unit (Millipore, Billerica, MA). Concurrently, Peptide-payload conjugate (**7**) was prepared by reacting 2mM aizoacetyl-Ser-Lys-Gly-Ser-Lys (CPC scientific, inc. Sunnywale, CA) with 8 mM NHS-ester payload [19] in 50% Dimethyl sulfoxide (DMSO), 50 mM borate buffer pH 8.5 for 2 h at 37°C. The excess NHS-ester payload was subsequently quenched by addition of 5 equiv of ethanolamine and incubation for 20 min at 37°C. Without further purification, this mixture containing 4 equiv of **7** was reacted with 5–10 mg/mL of **6** in DPBS for 3h at 37°C. The resulting MPP ADC was then purified and characterized as described above.

## Supporting information

S1 Checklist The ARRIVE guidelines checklist—Animal research. (PDF)

S1 Fig In vivo efficacy of thailanstatin MPP trastuzumab ADCs in N87 gastric cancer xenograft model. (A) In vivo efficacy of MPP ADC 13 and MPP ADC14 in N87 gastric cancer xenograft model dosed at 1.56 mg/kg (q4d x 4). (B) Individual mouse in vivo efficacy of MPP ADC13 in N87 gastric cancer xenograft model dosed at 3 mg/kg (q4d x 4) showing two complete regression. (C) Individual mouse in vivo efficacy of MPP **ADC14** in N87 gastric cancer xenograft model dosed at 3 mg/kg (q4d x 4) showing three complete regression.
(PDF)

S2 Fig In vivo efficacy of double-cysteine mutant thailanstatin trastuzumab ADC16 in N87 gastric cancer xenograft model. (A) Individual mouse in vivo efficacy of MPP-ADC 16 in N87 gastric cancer xenograft model dosed at 3 mg/kg (q4d x 4) showing three complete regression. (B) Body weight data in grams for mice treated with 3 mg/kg (q4d x 4) of **ADC16** in N87 efficacy model.
(PDF)

S3 Fig *In vivo* efficacy of T-DM1 in N87 gastric cancer xenograft model dosed at 1, 3 and 10 mg/kg (q4d x 4). (PDF)
