# Supplementary material for: Multivalent peptidic linker enables identification of preferred sites of conjugation for a potent thialanstatin antibody drug conjugate
Source: PLoS One. 2017 May 30;12(5):e0178452. doi: 10.1371/journal.pone.0178452 (PMC5448779; doi:10.1371/journal.pone.0178452)
Supplement: S1 Fig — (A) In vivo efficacy of MPP ADC 13 and MPP ADC14 in N87 gastric cancer xenograft model dosed at 1.56 mg/kg (q4d x 4). (B) Individual mouse in vivo efficacy of MPP ADC13 in N87 gastric cancer xenograft model dosed at 3 mg/kg (q4d x 4) showing two complete regression. (C) Individual mouse in vivo efficacy of MPP ADC14 in N87 gastric cancer xenograft model dosed at 3 mg/kg (q4d x 4) showing three complete regression. (PDF) [file pone.0178452.s002.pdf]

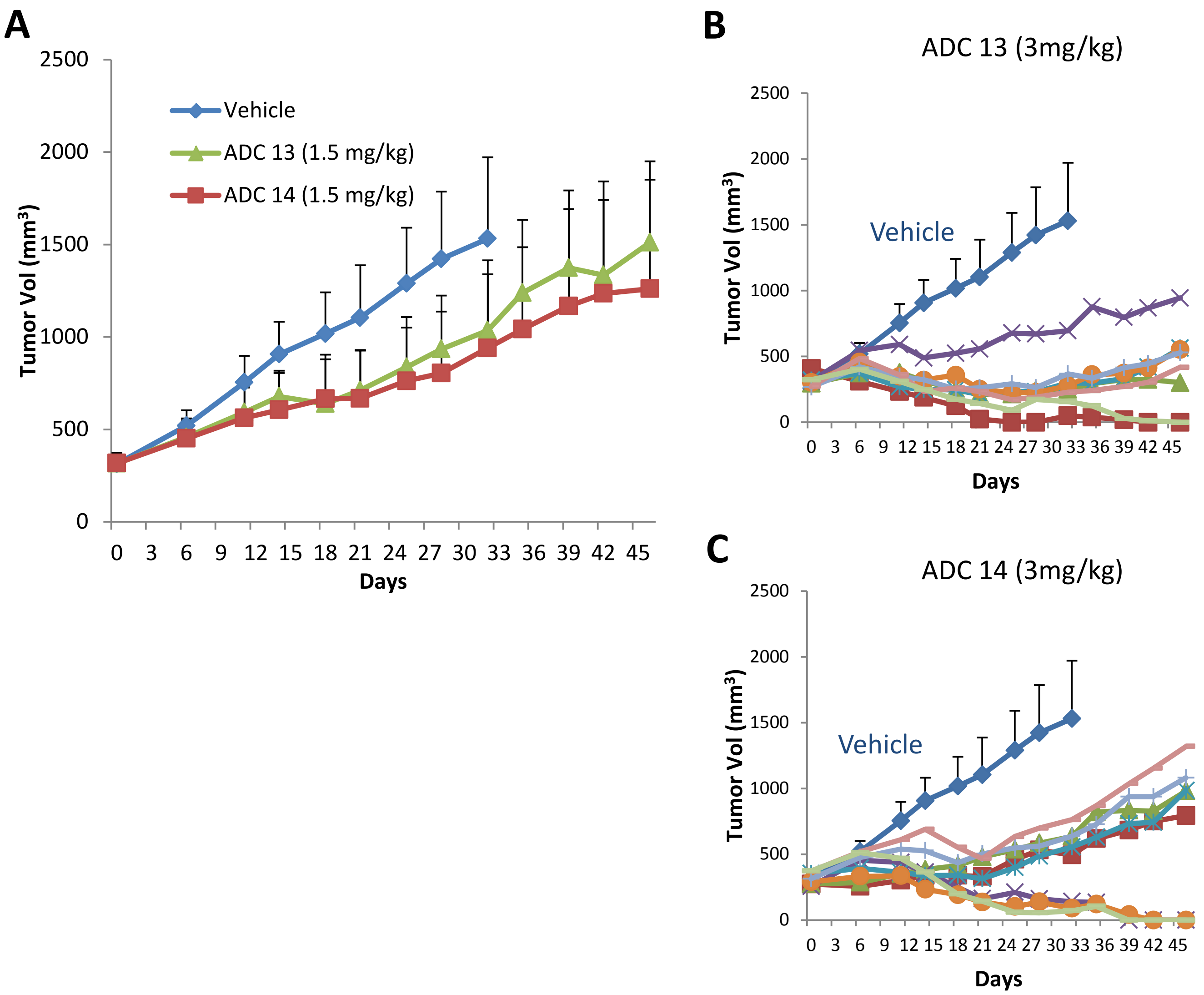

S1 Fig: In vivo efficacy of thailanstatin MPP trastuzumab ADCs in N87 gastric cancer xenograft model (A) In vivo efficacy of MPP-ADC 13 and MPP-ADC 14 in N87 gastric cancer xenograft model dosed at 1.56 mg/kg (q4d x 4). (B) Individual mouse in vivo efficacy of MPP-ADC 13 in N87 gastric cancer xenograft model dosed at 3 mg/kg (q4d x 4) showing two complete regression. (C) Individual mouse in vivo efficacy of MPP **ADC14** in N87 gastric cancer xenograft model dosed at 3 mg/kg (q4d x 4) showing three complete regression.
