# Supplementary material for: Multivalent peptidic linker enables identification of preferred sites of conjugation for a potent thialanstatin antibody drug conjugate
Source: PLoS One. 2017 May 30;12(5):e0178452. doi: 10.1371/journal.pone.0178452 (PMC5448779; doi:10.1371/journal.pone.0178452)
Supplement: S2 Fig — (A) Individual mouse in vivo efficacy of MPP-ADC 16 in N87 gastric cancer xenograft model dosed at 3 mg/kg (q4d x 4) showing three complete regression. (B) Body weight data in grams for mice treated with 3 mg/kg (q4d x 4) of ADC16 in N87 efficacy model. (PDF) [file pone.0178452.s003.pdf]

A

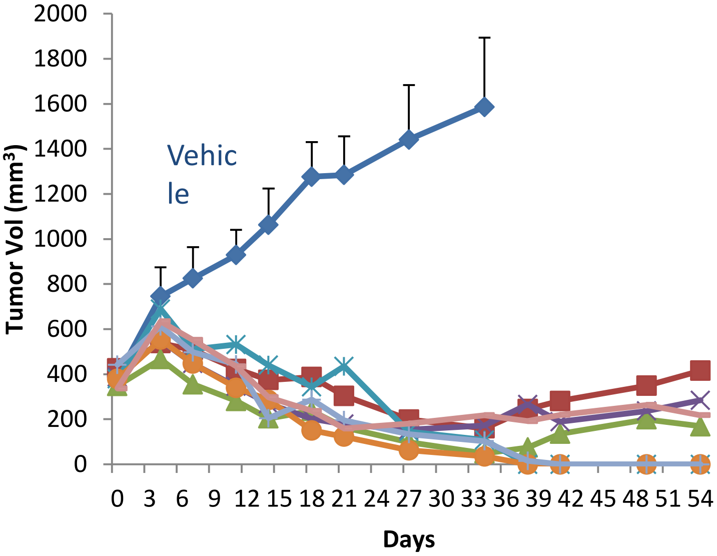

B

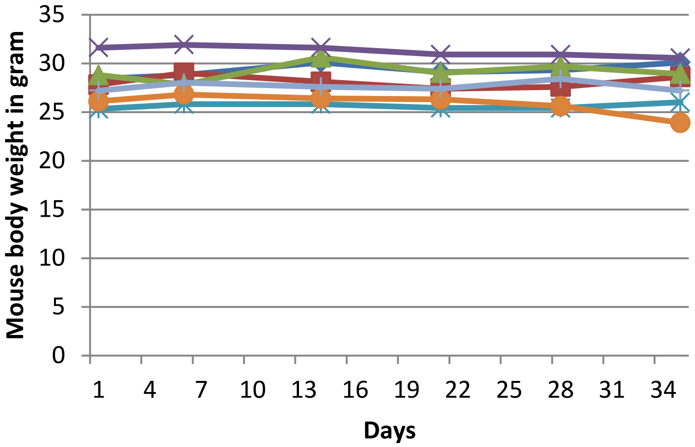

S2 Fig: In vivo efficacy of double-cysteine mutant thailanstatin trastuzumab ADC 16 in N87 gastric cancer xenograft model (A) Individual mouse in vivo efficacy of MPP-ADC 16 in N87 gastric cancer xenograft model dosed at 3 mg/kg (q4d x 4) showing three complete regression. (B) Body weight data in grams for mice treated with 3 mg/kg (q4d x 4) of **ADC16** in N87 efficacy model.
