# Supplementary figures and images for: Multivalent peptidic linker enables identification of preferred sites of conjugation for a potent thialanstatin antibody drug conjugate
Source: PLoS One. 2017 May 30;12(5):e0178452. doi: 10.1371/journal.pone.0178452 (PMC5448779; doi:10.1371/journal.pone.0178452)

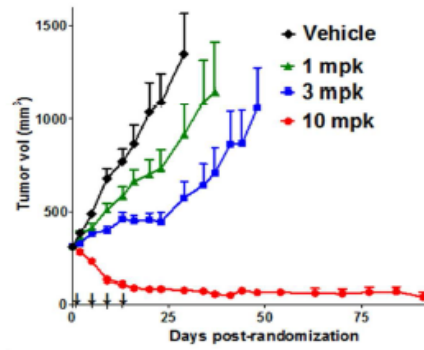

**S3 Fig:** *In vivo* efficacy of T-DM1 in N87 gastric cancer xenograft model dosed at 1, 3 and 10 mg/kg (q4d x 4).

Supplement: S3 Fig — (PDF) [file pone.0178452.s004.pdf]
